# Safety of Age-Adjusted D-dimer in Patients Aged ≥50 Years Undergoing Computed Tomography Pulmonary Angiogram (CTPA) for Suspected Pulmonary Embolism: A Retrospective Cohort Study

**DOI:** 10.7759/cureus.106475

**Published:** 2026-04-05

**Authors:** Isobel S Kelsh, Hadi Said, Nadeem Ijaz, Amina Kaddouri, Amy Oakes, Matthew King

**Affiliations:** 1 Acute Medicine, Western General Hospital, Edinburgh, GBR

**Keywords:** acute pulmonary embolism, age-adjusted d-dimer, computed tomography pulmonary angiography, false-negative, pre-test probability, pulmonary embolism (pe), retrospective cohort study, right ventricular heart strain

## Abstract

Aims

This study aims to assess the safety of age-adjusted D-dimer thresholds (AAT) and their impact on imaging utilisation for pulmonary embolism (PE) rule-out in settings where AAT are not routinely applied.

Methods and materials

We retrospectively studied patients aged ≥50 years who underwent D-dimer testing (without age adjustment) and computed tomography pulmonary angiogram (CTPA) for suspected PE at two different hospital sites (January 2022 to December 2023). Pre-test probability was recorded using the two-level Wells' score. Age-adjusted thresholds were retrospectively calculated, and sensitivity, specificity, negative predictive value (NPV), and failure rate were determined. False negatives were reviewed in further detail.

Results

Among 1,135 patients, PE was confirmed in 183 (16.1%). Age-adjusted D-dimer yielded 176 true positives, 7 false negatives, 755 false positives and 197 false negatives. Sensitivity was 96.2%, specificity 20.7% and NPV 96.6%. The failure rate among D-dimer negative patients was 3.4% (95% CI: 1.7-6.8%). False negatives included two subsegmental, three segmental with and without subsegmental elements, two lobar and one chronic embolus; none had right ventricular strain. Implementation of AAT could have avoided 197 CTPAs (17.4%), thereby reducing the imaging department's workload and unnecessary contrast and radiation administration.

Conclusion

In routine National Health Service (NHS) practice, age-adjusted D-dimer demonstrates high sensitivity and NPV in patients ≥50 years. The 3.4% failure rate was above the 3% benchmark, though all missed emboli were of low clot burden without right ventricular strain. These findings support integrating age-adjusted D-dimer into structured pathways to safely reduce CTPA use and resource burden.

## Introduction

Pulmonary embolism (PE) is a common and potentially fatal cardiovascular condition associated with significant morbidity and mortality worldwide. PE combined with deep vein thrombosis (DVT) makes up the third most common cardiovascular disease in the UK, accounting for 53-162 cases per 100,000 people, highlighting the importance of prompt recognition, accurate diagnosis and effective initial treatment to improve patient outcomes [1-3].

Computed tomography pulmonary angiogram (CTPA) remains the gold standard for diagnosing PE, but its use has increased substantially over the past decade. A large cohort study in America reported a 450% rise in CTPA use from 2004 to 2016, raising concerns about overuse, unnecessary radiation exposure and pressure on imaging resources [4]. In the UK, audits show that most patients with suspected PE undergo CTPA. Yet, positivity rates are often below 20%, meaning the majority of scans are negative for PE but often pick up alternative diagnoses, most commonly infection or malignancy [5,6].

To improve diagnostic efficiency, emergency and acute medicine departments rely on a stepwise approach when faced with a patient with a suspected PE, which combines pre-test probability (PTP) and serum D-dimer testing. The two-level Wells' score stratifies patients as either “PE unlikely" or “PE likely" [7]. Patients with low or moderate PTP (“PE unlikely”) undergo serum D-dimer testing. A D-dimer result below a conventional threshold (<250 µg/L) effectively rules out PE without the need for further cross-sectional imaging. However, patients with a high PTP (“PE likely”) proceed directly to CTPA, bypassing serum D-dimer analysis. Similar to the two-level Wells' Score, the Simplified Geneva score [8] is another PTP system that can be applied once clinical suspicion of PE is raised. The application of these pathways aims to reduce unnecessary imaging, limit radiation and contrast exposure, optimise resource use and help guide clinician workup.

D-dimer, a fibrin degradation product, is highly sensitive for venous thromboembolism (VTE) but has low specificity, and elevated levels may also occur in cancer, recent surgery, inflammation, pregnancy, and, notably, aging [9]. Specificity declines with increasing patient age, contributing to the rising proportion of negative CTPAs in older adults. Age-adjusted D-dimer thresholds (AAT) have been proposed to improve specificity in patients over the age of 50, potentially reducing unnecessary imaging. While several studies suggest that the application of AAT is safe and effective [10,11], its implementation in routine practice requires robust evidence to ensure adequate patient safety.

As AAT are not routinely applied in most hospitals, including those in this study, we aimed to evaluate the safety of applying AAT in patients with suspected PE in routine National Health Service (NHS) practice and to quantify their impact on imaging utilisation.

## Materials and methods

Study design and setting

This was a retrospective cohort study conducted across two hospital sites, with data collected over a two-year period from January 2022 to December 2023. The study included patients aged ≥50 years who underwent CTPA for suspected PE.

Data sources

Clinical and imaging report data were retrieved from electronic health record systems (TRACKcare) and radiology imaging systems (Picture Archiving and Communication System (PACS)).

**Figure 1:**
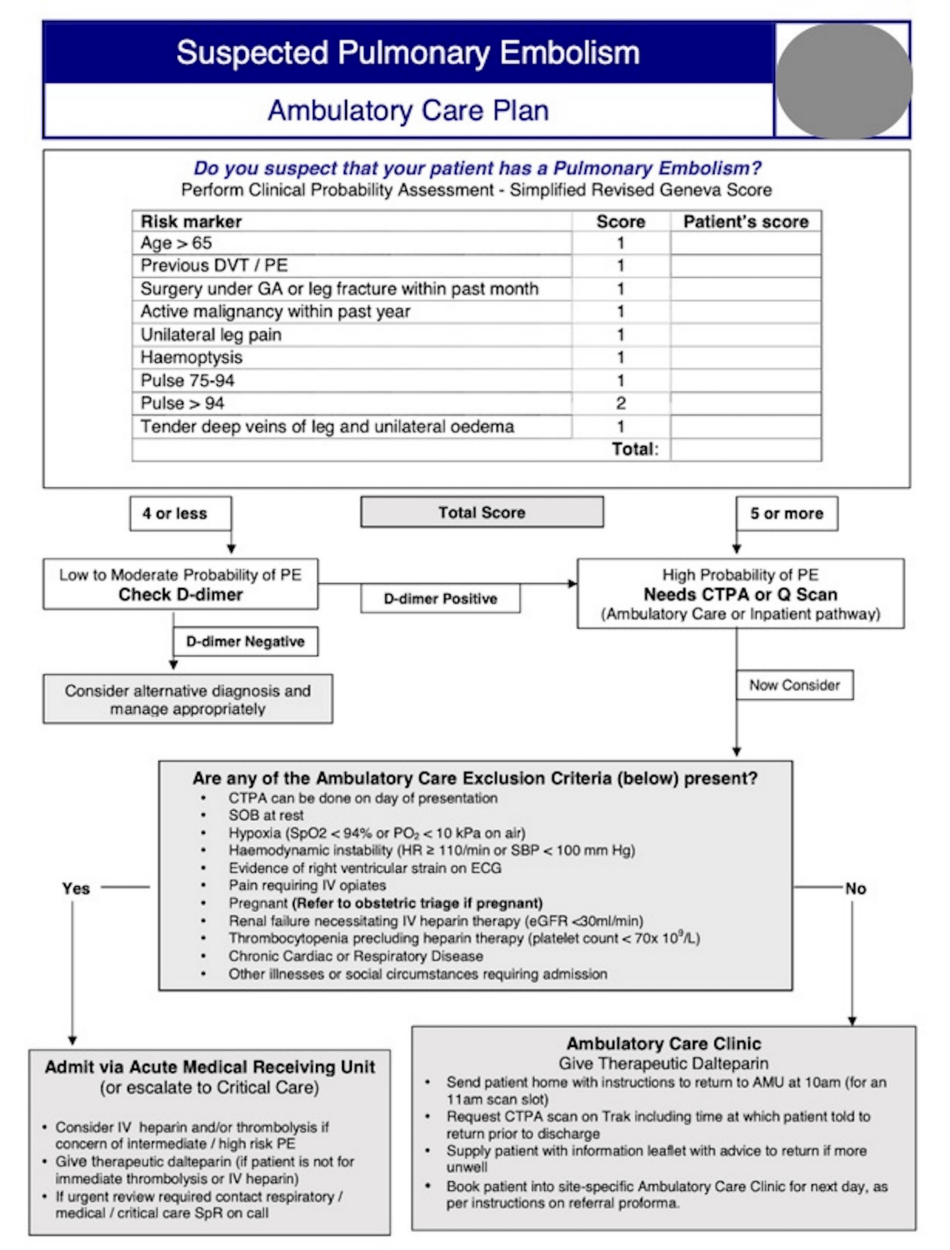
Ambulatory care pathway for suspected pulmonary embolism. This figure demonstrates the clinical decision-making pathway in acute medicine within this cohort's hospital trust. The clinical pathway invites the clinician to apply pre-test probability in the form of the Simplified Geneva Score, similar to the Wells' two-level score, in conjunction with serum D-dimer results. DVT: deep vein thrombosis; PE: pulmonary embolism; CTPA: computed tomography pulmonary angiogram; GA: general anaesthesia.

PTP was intended to be assessed using the local protocol, which suggested the use of the simplified Geneva score. However, during data collection, it became apparent that this protocol was not consistently applied by clinicians. Some clinicians used the two-level Wells' Score (“PE unlikely”/“PE likely”), but its application was also variable and not standardised across cases. As this was a retrospective study, PTP assessment reflected routine clinical practice rather than a uniform protocol. An example of a site-specific PTP diagnostic pathway is shown in Figure 1.

CTPA studies were reported by Radiology Registrars or Consultant Radiologists in accordance with standard departmental reporting procedures.

Data extraction was performed by all authors and distributed evenly across the study cohort. Prior to data collection, a standardised data entry framework was developed and implemented in Microsoft Excel (Microsoft Corporation, Redmond, WA) to ensure consistency and uniformity in data recording. A subset of cases was independently cross-checked between authors to ensure accuracy and consistency of data extraction. Extracted data were reviewed for completeness, and cases with missing key variables (e.g., D-dimer results) were excluded as described. No imputation of missing data was performed.

Patient selection

**Figure 2:**
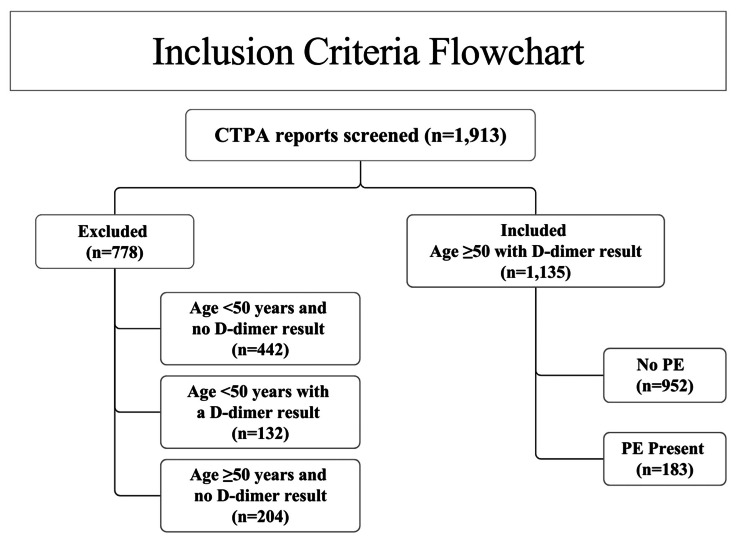
Flowchart of inclusion and exclusion criteria. This flowchart demonstrates the initial pool of patient computed tomography pulmonary angiogram (CTPA) reports, n=1913. Exclusion criteria were set to remove those requests where the patients were aged <50 years and did not undergo D-dimer serum analysis. These exclusions removed 779 patients from the dataset, leaving a total of 1135 patients for analysis. Of these 183 CTPA reports were found to be positive for pulmonary embolism (PE).

Patients were excluded if they were <50 years of age or if no D-dimer testing had been performed (n=778). After applying these exclusions, 1135 patients made the final cohort, see Figure 2.

Follow-up data were obtained in April 2025 to assess patient outcomes and the safety of the potential age-adjusted rule-out.

D-dimer measurements

D-dimer assays were reported in D-dimer units (DDU), with the conventional positive threshold set at 250 µg/L. AAT were calculated retrospectively using the formula: age × 5 µg/L (e.g., for a patient aged 90 years, the AAT cut-off was 450 µg/L) [12].

Outcome definitions

The primary endpoint was the false negative rate, defined as the proportion of patients classified as negative by AAT who were subsequently found to have a CT-confirmed PE. According to international guidance on PE rule-out strategies, a failure rate of <3% is considered acceptable for safe exclusion of clinically significant PE [1,7].

Statistical analysis

Diagnostic analysis metrics were calculated, including sensitivity, specificity, NPV and failure rate. The proportion of missed PE cases was determined with an approximate 95% confidence interval, using Wilson's method. Continuous variables are presented as mean ± standard deviation or median with interquartile ranges, as appropriate. Categorical variables are reported as counts and percentages.

## Results

Cohort characteristics

**Figure 3:**
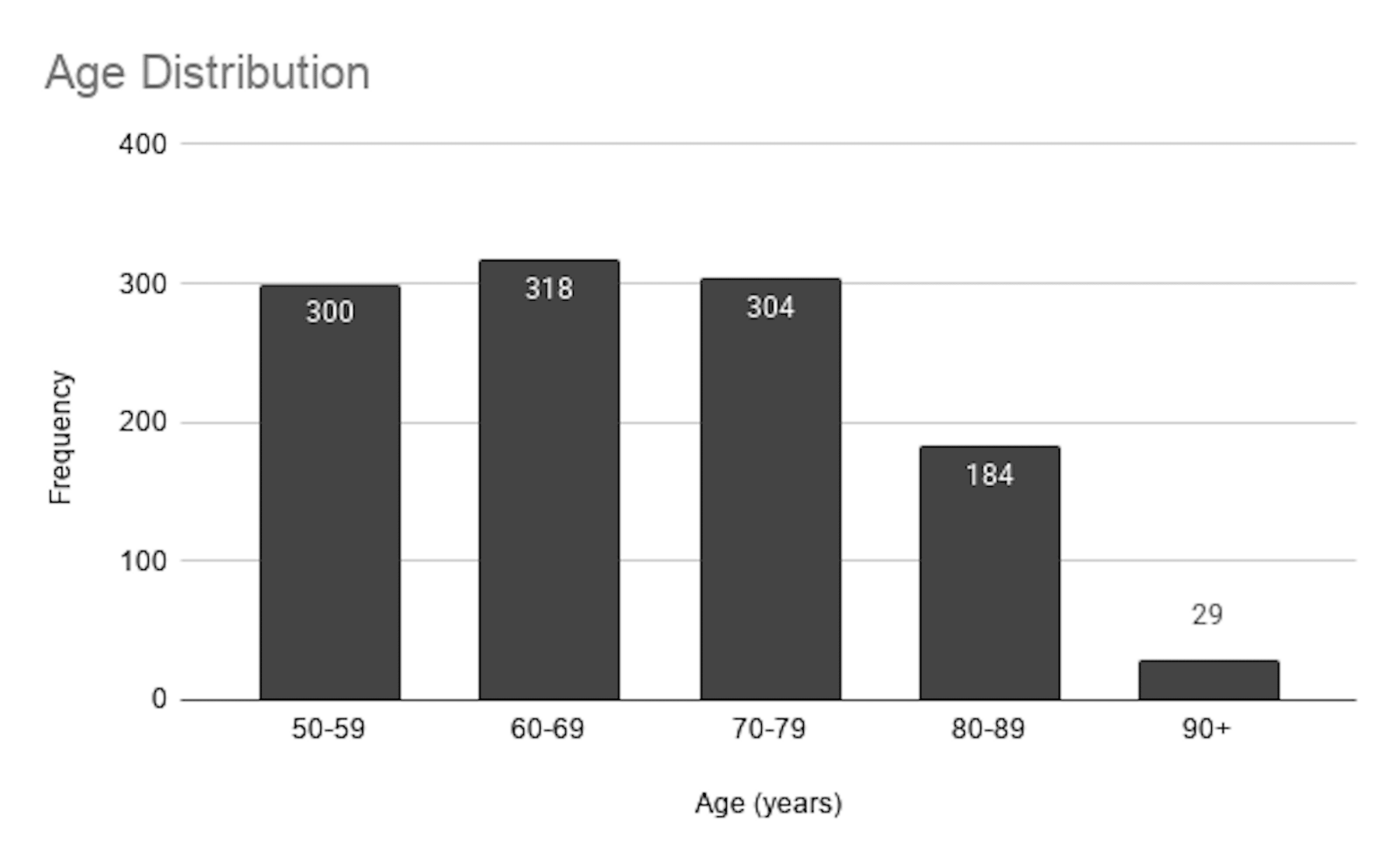
Bar chart to show age distribution for the included patient cohort. This Bar chart shows the distribution in age of the included patient cohort. Most patients were aged 50-79 years, with the highest frequency observed in the 60-69 age group, followed closely by the 70-79 age group and those aged 50-59 years. Smaller numbers were observed in the 80-89 age group and those aged ≥90 was the smallest.

A total of 1,135 patients aged ≥50 years who underwent both D-dimer testing and CTPA for suspected PE were included in the final analysis. Most patients were aged 50-79 years, with the highest frequency observed in the 60-69 age group (n=318, 28%), followed closely by the 70-79 age group (n=304, 26.8%) and those aged 50-59 years (n=300, 26.4%). Smaller numbers were observed in the 80-89 age group (n=184, 16.2%) and those aged ≥90 was the smallest (n=29, 2.6%), see Figure 3.

**Figure 4:**
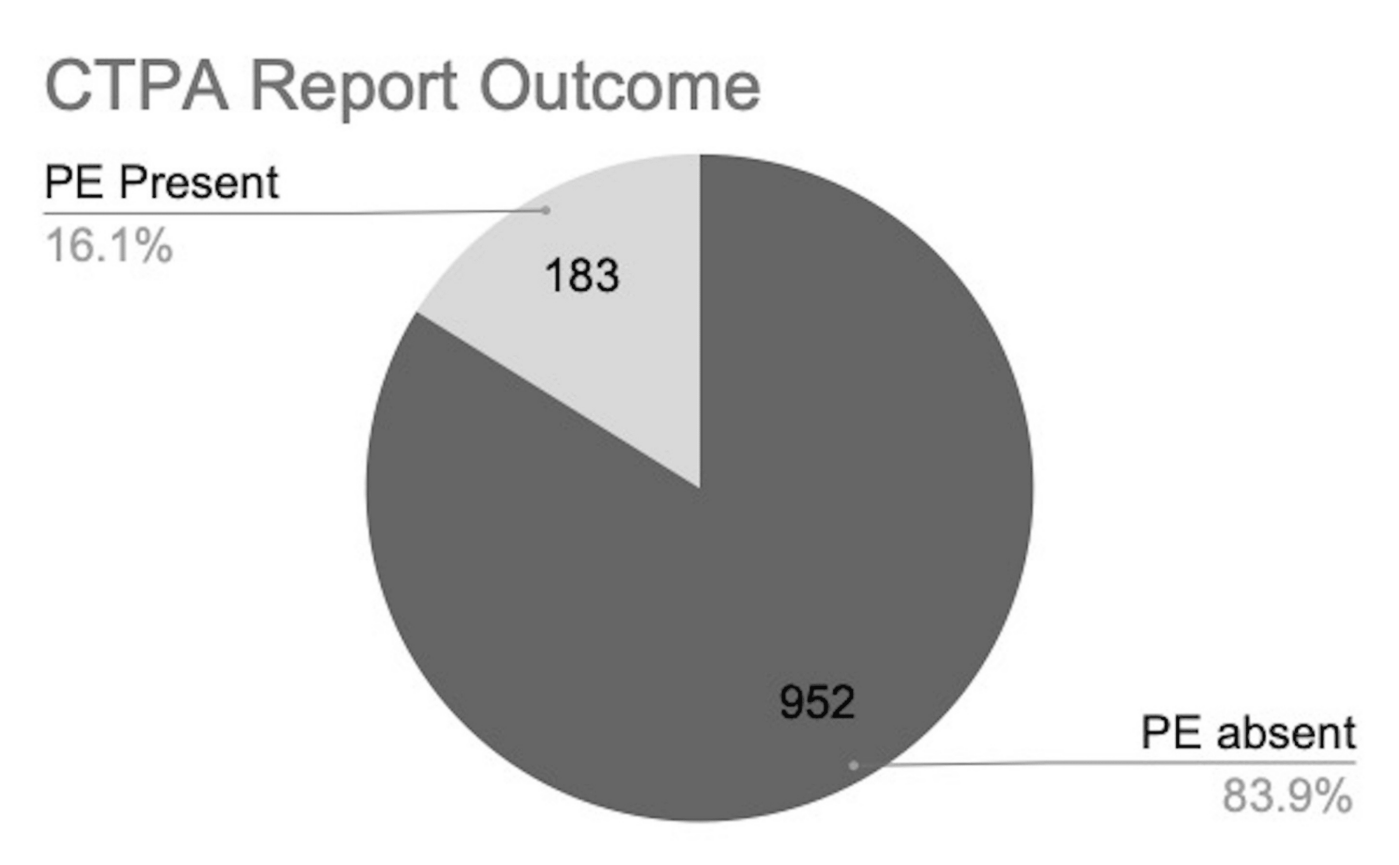
Pie chart demonstrating CTPA report outcome. This pie chart shows that 183 patients (16.1%) were found to have CT-confirmed PE, while 952 (83.9%) had no evidence of PE on CTPA. CTPA: computed tomography pulmonary angiogram; PE: pulmonary embolism.

PE was confirmed in 183 patients (16.1%), while 952 (83.9%) had no evidence of PE on CTPA, see Figure 4.

Diagnostic performance of the standard D-dimer threshold

Using the standard D-dimer threshold, 183 patients (16.1%) with PE had a positive serum result, and there were no false negatives identified. There were 916 false positives and 36 true negatives, yielding a sensitivity of 100% (95% CI: 98.4-100%) and specificity of 3.8% (95% CI: 2.6-5.0%). The positive and negative predictive values were 17.6% (95% CI: 14.5-18.9%) and 100% (95% CI: 91.7-100%), respectively.

Diagnostic performance of age-adjusted D-dimer

Application of AAT resulted in 176 (15.5%) true positives, 7 (0.6%) false negatives, 755 (66.5%) false positives and 197 (17.4%) true negatives, see Table 1.

**Table 1:** Age-adjusted D-dimer classification compared with CTPA-confirmed pulmonary embolism. This table demonstrates the classification of CTPA outcomes. Application of AAT resulted in 176 (15.5%) true positives, 7 (0.6%) false negatives, 755 (66.5%) false positives and 197 (17.4%) true negatives. AAT: age-adjusted D-dimer threshold; CTPA: computed tomography pulmonary angiogram; PE: pulmonary embolism.

	PE present	PE absent	Total
Age-adjusted D-dimer positive	176	755	931
Age-adjusted D-dimer negative	7	197	204
Total	183	952	1135

Sensitivity was 96.2%, specificity 20.7% and NPV 96.6%. The failure rate among negative D-dimer patients was 3.4% (95% CI: 1.7-6.8%), with 3.8% of all PE cases missed. Compared with the standard threshold, age-adjustment increased specificity from 3.8% to 20.7%, reducing false positives by 161 cases. This occurred alongside a reduction in sensitivity from 100% to 96.2%, see Table 2.

**Table 2:** Diagnostic performance of standard vs age-adjusted D-dimer thresholds. PE: pulmonary embolism.

Measure	Standard D-dimer cut-off (<250 µg/L)	Age-adjusted D-dimer threshold (AAT)
True positives (TP)	183	176
False negatives (FN)	0	7
False positive (FP)	916	755
True negatives (TN)	36	197
Sensitivity (%)	100	96.2
Specificity (%)	3.8	20.7
Negative predictive value (%)	100	96.6
Positive predictive value (%)	16.7	18.9
Failure rate (%)	0	3.4
Proportion of all PE cases missed (%)	0	3.8

Characterisation and Analysis of False Negative Cases

The seven false negative cases were examined in further detail. The ages of these patients ranged from 61 to 90 years, with a median age of 66. D-dimer values ranged from 252 to 342 µg/L DDU. In four cases, D-dimer values were within 11 µg/L of the AAT, see Table 3.

**Table 3:** Analysis of false negative cases. This table illustrates in more detail the seven false negative cases. The ages of these patients ranged from 61 to 90 years, with a median age of 66. D-dimer values ranged from 252 to 342 µg/L DDU. In four cases, D-dimer values were within 11 µg/L of the AAT. DDU: D-dimer units; PE: pulmonary embolism.

Age (years)	Actual D-dimer (µg/L DDU)	Age-adjusted D-dimer threshold (AAT) (µg/L DDU)	Difference between AAT and actual D-dimer result (µg/L DDU)	Wells’ criteria score (risk)	PE classification
77	326	385	59	Moderate	Chronic, no right heart strain
63	270	315	45	Not documented	Multiple, right upper, middle and lower lobes, no right heart strain
90	342	450	108	Moderate	Small volume, right upper lobe, no right heart strain
66	321	330	9	Not documented	Low volume, left lower lobe, no right heart strain
65	314	325	11	Not documented	Low volume, left lower lobe, no right heart strain
77	252	385	133	Moderate	Small volume, left upper lobe, no right heart strain
61	300	305	5	Not documented	Low volume, left lower lobe, no right heart strain

Radiological characterisation demonstrated two isolated subsegmental emboli, three segmental emboli with or without additional subsegmental involvement and two lobar emboli. One case was interpreted as chronic thromboembolic disease rather than acute PE. In all cases, there was no CT evidence of right ventricular strain. Follow-up analysis to April 2025 demonstrated that six of the patients were alive. One patient was deceased, the Medical Certificate of Cause of Death was unavailable, and causality could not be demonstrated.

Potential reduction in imaging using age-adjusted D-dimer

Of the 952 patients with negative CTPA reports for PE, 197 (20.7%) had a negative age-adjusted D-dimer result and could have potentially avoided imaging, representing 17.4% of all CTPAs performed. Furthermore, 36 (3.8%) of these 952 patients underwent imaging despite having D-dimer values below the conventional cutoff of 250 µg/L. From analysis of the TRACKcare entry data, these scans were performed based on ongoing clinical concern for PE. Documented application of appropriate PTP (calculation of two-point Wells' scores) was identifiable from clinical data entry or CTPA radiology requests in only 99 patients (8.7%) of the total included study cohort.

## Discussion

In this retrospective study of 1,135 patients undergoing CTPA for suspected PE, application of AAT demonstrated a high level of safety, with a sensitivity of 96.2% and a NPV of 96.6%. Implementation of AAT would have avoided 197 CTPAs (17.4% of all scans), while only missing 7 PE cases (3.8% of all PE). Although this observed failure rate lies marginally above the conventionally cited 3% safety threshold for PE exclusion, the confidence interval includes values below this benchmark, reflecting statistical uncertainty and the relatively small number of false negative events. The impactful trade-off for the reduction in CTPA use was a small number of radiologically detected emboli, all of which carry a low clot burden and without ventricular strain. In an era of increasing CTPA demand within emergency and acute medicine departments, strategies that safely reduce pressure on imaging departments are of clear service and patient benefits. These findings indicate that age-adjusted D-dimer can safely be used to reduce unnecessary imaging while maintaining a high probability of ruling out PE.

This study cohort reflects real-world NHS practice and included patients who proceeded to imaging. According to Royal College of Radiology (RCR) guidance, the expected diagnostic yield of CTPA in suspected PE is approximately 15-37%, placing our study cohort with a PE prevalence 16.1%, at the lower end of the recommended range [5]. A detailed review of the seven false negative cases provides important clinical context. All emboli were of low clot burden, being subsegmental or lobar in distribution. Furthermore, no patient demonstrated radiological evidence of right ventricular strain. Three cases where the actual recorded D-dimer result lay maximally 11 µg/L within the calculated AAT suggest proximity to the diagnostic cutoff rather than marked biological discordance. These cases would likely have proceeded to CTPA given the small difference in cut-off, so these three patients might not have been missed and counted as a false negative in applied practice.

Our findings are consistent with other validation studies of age-adjusted D-dimer. The ADJUST-PE prospective multicentre outcome study demonstrated that using an age-adjusted cut-off (age x 10 µg/L in patients ≥50 years) in fibrinogen equivalent units increased the proportion of patients in whom PE was excluded without imaging, with a similarly low failure rate at three-month follow-up [11]. Other retrospective studies have also shown that age-adjustment can reduce unnecessary imaging in older adults whilst maintaining an acceptable sensitivity [13]. Furthermore, other research supports that age-adjusted thresholds reduce false positives compared with standard thresholds, leading to potentially large reductions in imaging use [14]. The modest reduction in sensitivity observed in our audit aligns with prior trials, reflecting the expected trade-off between increasing specificity and a slight reduction in sensitivity. Differences in absolute values may be expected by variations in patient demographics, local D-dimer assays and CTPA ordering patterns. Overall, our results support the safety and effectiveness of AAT in real-world clinical practice.

From an imaging service perspective, applying AAT has multiple benefits. Avoiding 17.4% of CTPAs would hugely reduce an already overloaded scanner workload, decreasing patient exposure to intravenous contrast and ionising radiation, and improving overall service efficiency. This is especially important in high-volume centres, where optimising scanner capacity and minimising reporting lists are key operational priorities. These factors are even more relevant to consider in older adults undergoing CTPA, in whom incidental findings are more common, often prolonging radiologist's reporting times and necessitating sometimes multiple additional follow-up investigations or further interval surveillance imaging.

This study has several strengths, including its two-centre design within a large NHS health board, real-world data capture, detailed radiological characterisation of false negative cases and extended follow-up. This audit reflects real-world, consecutive cohorts of patients undergoing CTPA for suspected PE, enhancing the generalisability of our findings. All patients had complete follow-up data, ensuring accurate collection of missed PE cases. The study design allowed direct comparison between standard and age-adjusted D-dimer strategies using the same patient cohort, strengthening the internal validity of the analysis.

However, limitations must also be acknowledged. As only patients who underwent CTPA were included, the true diagnostic performance of age-adjusted D-dimer in the broader suspected PE population cannot be fully determined. Confidence intervals around the failure rate are relatively wide due to a smaller number of false negative events. Additionally, the cause of death data was not available for one patient, limiting the definitive assessment of PE-related mortality. This was a retrospective, two-centre audit, limiting generalisability, introducing potential selection bias. Clinical probability scoring was not systematically applied, which could influence D-dimer performance. The clinical pathways for the included study hospital departments recommend the use of simplified Geneva score; however, the PTP applied in any case was either a two- or three-level Wells' score. A documented Wells' score was identifiable in only 99 patients (8.7%), and only 50.5% of these were applied correctly, e.g., a “PE unlikely” PTP score required a D-dimer assay, a “PE likely” score negated the need for D-dimer analysis before CTPA scanning. The limited application of PTP in this cohort reflects real-world practice but may influence the observed performance of D-dimer testing. Differences in local D-dimer assays and cut-offs may limit direct extrapolation to other hospitals. Despite these limitations, the study provides valuable real-world insight into the impact of age-adjusted D-dimer on imaging utilisation and patient safety.

## Conclusions

Age-adjusted D-dimer demonstrates high sensitivity and negative predictive value in patients aged ≥50 years investigated for suspected PE in routine clinical practice. Although the observed failure rate was slightly above the conventional 3% benchmark, missed events were of low clot burden and occurred without evidence of cardiac compromise. Implementation of age-adjusted D-dimer testing could reduce unnecessary CTPA use, thereby decreasing radiation exposure, contrast use, scanner and radiologists’ workloads, particularly in older adults. These findings support the safe integration of age-adjusted D-dimer into structured diagnostic pathways, with careful patient selection and adherence to validated pre-test probability assessment.
